# Gene expression profiles during early differentiation of mouse embryonic stem cells

**DOI:** 10.1186/1471-213X-9-5

**Published:** 2009-01-09

**Authors:** Fiona C Mansergh, Carl S Daly, Anna L Hurley, Michael A Wride, Susan M Hunter, Martin J Evans

**Affiliations:** 1 School of Biosciences, Cardiff University, Museum Avenue, Cardiff, CF10 3US, Wales, UK; 2 Ocular Genetics Unit, Smurfit Institute of Genetics, Trinity College Dublin, Dublin 2, Ireland; 3 Zoology Department, Trinity College Dublin, Dublin 2, Ireland

## Abstract

**Background:**

Understanding the mechanisms controlling stem cell differentiation is the key to future advances in tissue and organ regeneration. Embryonic stem (ES) cell differentiation can be triggered by embryoid body (EB) formation, which involves ES cell aggregation in suspension. EB growth in the absence of leukaemia inhibitory factor (LIF) leads EBs to mimic early embryonic development, giving rise to markers representative of endoderm, mesoderm and ectoderm. Here, we have used microarrays to investigate differences in gene expression between 3 undifferentiated ES cell lines, and also between undifferentiated ES cells and Day 1–4 EBs

**Results:**

An initial array study identified 4 gene expression changes between 3 undifferentiated ES cell lines. Tissue culture conditions for ES differentiation were then optimized to give the maximum range of gene expression and growth. -Undifferentiated ES cells and EBs cultured with and without LIF at each day for 4 days were subjected to microarray analysis. -Differential expression of 23 genes was identified. 13 of these were also differentially regulated in a separate array comparison between undifferentiated ES cells and compartments of very early embryos. A high degree of inter-replicate variability was noted when confirming array results. Using a panel of marker genes, RNA amplification and RT-PCR, we examined expression pattern variation between individual -D4-Lif EBs. We found that individual EBs selected from the same dish were highly variable in gene expression profile.

**Conclusion:**

ES cell lines derived from different mouse strains and carrying different genetic modifications are almost invariant in gene expression profile under conditions used to maintain pluripotency. Tissue culture conditions that give the widest range of gene expression and maximise EB growth involve the use of 20% serum and starting cell numbers of 1000 per EB. 23 genes of importance to early development have been identified; more than half of these are also identified using similar studies, thus validating our results. EBs cultured in the same dish vary widely in terms of their gene expression (and hence, undoubtedly, in their future differentiation potential). This may explain some of the inherent variability in differentiation protocols that use EBs.

## Background

ES cells are derived from the inner cell masses of blastocysts and can contribute to all cell types of the embryo proper [1,2]. The combination of pluripotency and ease of genetic modification has given rise to the revolution in genetic analysis via the use of knockout mice. ES cell pluripotency has also been exploited *in vitro*; many different cell types can now be generated in culture. Human ES cell lines have been isolated; moreover, ES like cells (iPS cells) can be derived from human skin [3-6]. Therefore, *in vitro *differentiation protocols for human ES or iPS cells have huge therapeutic potential.

Many in vitro ES differentiation protocols rely on embryoid bodies (EBs); floating aggregates of ES cells which, when grown without LIF, mimic to some extent the early stage embryo, giving rise to precursors of a large number of tissues[7,8]. Early growth of EBs with LIF favours stem cell renewal and the differentiation of embryonic endoderm [9], while removal of LIF allows the generation of precursors representative of all three germ layers [10]. To date, protocols for deriving neural, haematopoietic, muscle, bone, pancreatic, hepatic and many other precursor and mature cell types from ES cells in culture [11-13] have been developed, many of which still use EBs as a starting point. EBs can be allowed to differentiate spontaneously from cell suspension or can be formed from a defined cell number using 'hanging drops'. Following aggregation, culture is often allowed to proceed spontaneously for 3–4 days, followed by the addition of factors that promote differentiation of specific precursor types; for example, retinoic acid may be added to promote neuronal specification [14,15], although, today, more efficient neural differentiation can be achieved in chemically defined medium or via adherent monoculture in the presence of FGF [16-18]. Subsequent growth, followed by disassociation and plating on adherent surfaces, permits the derivation of terminally differentiated cell types.

ES differentiation can provide abundant, partially synchronised sources of transient embryonic precursor types that are present only in very limiting quantities *in vivo*. Moreover, EBs represent a good model for examining the events of early embryogenesis, as the formation of a pro-amniotic cavity and the expression of markers of early differentiation, for example, are often mimicked by EBs [19]. However, final cell populations are usually heterogeneous, percentages of desired cell types arising often vary from one experimental replicate to another [14,15,10]. Reasons for this variation are not hard to identify; differentiation is sensitive to glucose concentration, serum quality, amino acids, growth factors, extracellular matrix proteins, pH, osmolarity, passage number and the identity of the ES cell line used [13]. The existence of ES cell derived chimeras and ensuing mouse lines demonstrates the ability of ES cells to differentiate into all adult cell types. However, we are at present unable to generate the full complement of adult cell types from ES cells *in vitro*. Greater understanding of gene expression during early differentiation may allow more precise direction of ES cell differentiation and will also widen understanding of early embryonic development.

Dissecting the events of early differentiation has been aided by the development of microarray technology, which allows the examination of global gene expression changes. We have used microarray technology to examine variation between 3 undifferentiated ES cell lines. We then optimised aggregation methods, EB size, and serum concentrations and carried out array analysis using day 1–4 EBs in the presence and absence of LIF. We identified 23 differentially regulated genes, some of which have known roles in early development. However, given lower than expected confirmation rates, and lack of reproducibility in stem cell derived arrays [20-22], we tested the replicability of gene expression patterns arising from individual EBs growing in the same culture, using RNA amplification. We found a high level of variation in gene expression patterns, even between EBs from the same culture dish. This variability may provide explanations for the difficulties involved in obtaining pure cultures of differentiated progeny from EB based protocols.

## Methods

### Tissue Culture and cell lines

This study utilised 3 ES cell lines, IMT11 (derived from 129 mice), HM1 (which is Hprt negative) and SMHBl6 (derived from C57Bl6/J mice). The IMT11 line was selected for all investigations involving differentiation, as it is not genetically modified and is better characterised than SMHBl6. IMT11 cells showed the highest percentage of diploid cells after karyotyping and have been tested for germline transmission.

1) Undifferentiated ES cells were maintained at 37°C in a humidified atmosphere with 5% CO_2 _on 0.1% gelatin in DMEM, with 2 mM L-glutamine, 50 U/ml penicillin, 50 μg/ml streptomycin (all from Gibco™, Invitrogen Ltd, Paisley, Renfrewshire, UK), 10^-4 ^M β-Mercaptoethanol (Merck KGaA, 64293 Darmstadt, Germany), 10^-3 ^U/ml murine LIF (ESGRO™, Invitrogen, Ltd, Paisley, Renfrewshire, UK), 10% FBS (foetal bovine serum) and 10% NBS (newborn bovine serum) (selected batches, PAA Laboratories GmbH, Linz, A-4020 Austria). All undifferentiated ES cell lines were karyotyped using standard protocols in order to test that the majority of cells showed a normal diploid chromosome number (40XY) prior to differentiation.

2) EB generation: a semi-confluent 100 mM dish of ES cells was trypsinized (0.25% trypsin/EDTA, Invitrogen), followed by trituration in additional ES medium to achieve a single cell suspension. ES medium was prepared as above for + LIF EBs, and without LIF for -LIF differentiations. Cells were counted using a haemocytometer and cell density was adjusted appropriately to the required number of cells per 10 μl. A multichannel pipette (Finnpipette 5–50 μl) was used to deposit approximately 200 10 μl drops on the floor of a 140 mm bacteriological dish (Sterilin). A smaller plate was filled with 1–2 mls PBS and placed in the lid of the bacteriological dish. The plate was inverted and incubated overnight to allow the EBs to aggregate. The following day dishes were righted and flooded with 20 mls of the appropriate differentiation medium, then grown in suspension culture until harvesting. EBs were formed by aggregation of 125, 250, 500, 750 and 1000 cells per 10 μl to determine optimal size. A 50% FCS, 50% NCS mix was prepared from ES batch tested serum samples (PAA), then added to serum free ES medium, at 0%, 5%, 10%, 15% and 20% final volumes. Hanging drops containing either 750 or 1000 cells were generated in order to test serum concentrations.

We also tested EBs that were allowed to aggregate randomly following dissociation. For brevity, we refer to these as "random EBs later in the text". Random aggregation gives rise to greater numbers of EBs per dish, that are of much more variable sizes and shapes than those generated using the "hanging drop" method. For random aggregation of EBs, 1 × 10^6 ^cells were suspended in 10 mls media in a 100 mm bacteriological dish and allowed to aggregate spontaneously. Serum quantities were varied as above. Photographs were taken of EBs at day 4 of differentiation without LIF, at varying sizes and serum concentrations and saved in .tif format. Photographs were analysed and EB diameters measured using Scion Image (Scion Corporation). Diameter measurements were used to calculate radius and volume.

### RNA extraction and amplification

1) For array analysis, undifferentiated ES cells were washed twice with DPBS-A, treated with trypsin-EDTA (Invitrogen Ltd., Paisley, UK) and counted. Appropriate cell numbers were pelleted by centrifugation at 1000 g. The QIAgen RNeasy™ Midi Kit (Qiagen Ltd., Crawley, Sussex, UK) was used according to the manufacturer's protocol for RNA extraction, followed by OD 260/280 spectrophotometry (Camspec, Cambridge, UK) and gel electrophoresis using dissociating conditions (NorthernMax™ buffers; Ambion, Huntingdon, UK), used according to the manufacturer's protocol, to check RNA concentration and integrity.

2) For RNA extraction from individual embryoid bodies, the mini RNA isolation kit (Zymo) was used, followed by amplification using the RNA amplification kit (Arcturus), both according to the manufacturer's protocol. Following quantification by UV spectrophotometer (Camspec), amplified RNA samples were DNase treated using the Turbo DNAfree kit (Ambion) and reverse transcribed using the random hexamer protocol of the Superscript First Strand Synthesis System for RT-PCR (Invitrogen). (Note, a nuclease step is included in the Arcturus kit to remove residual DNA, but is not entirely sufficient to ensure blank negative control lanes, hence the extra DNase step).

3) For RNA extraction followed by RT-PCR, embryoid bodies were spun down, washed with PBS, then spun down again. EBs were treated with 0.25% trypsin/EDTA and washed with PBS a second time when extracting RNA from larger Day 3–4 EBs 100 ul of EBs+ residual PBS was resuspended in 1 ml TRIzol (Invitrogen); samples were homogenized by pipetting up and down using a 1 ml micropipette. RNA was extracted according to the manufacturer's protocol, quantified, DNase treated and reverse transcribed as above, except that the oligo-dT supplied with the kit was spiked with a 1/10000 dilution of an 18S rRNA gene specific primer, in order to allow 18S rRNA (which doesn't have a poly A tail) to be used as a housekeeping control. Where starting RNA quantity was low, we tripled the amount of RNA used and therefore the reaction volumes per sample. RT reactions were diluted with nuclease free water (Ambion) to 50 ul before PCR analysis. A "no RT" control corresponding to each sample was also produced for all RT-PCR experiments described in this paper; these were treated in exactly the same way as the samples except that reverse transcriptase was not added.

4) For RNA extraction prior to array analysis, EBs were spun down, washed, and trypsinized as above. The QIAgen RNeasy™ Midi Kit (Qiagen Ltd., Crawley, Sussex, UK) was used according to the manufacturer's protocol for RNA extraction. RNA samples were quantified and checked for quality as described in 1) above.

### Cy labelling for array analysis

10 μg of total RNA was labelled with either Cy3 or Cy5 dyes using the CyScribe labelling system (GE Healthcare, Chalfont St. Giles, Bucks, UK), according to the manufacturer's protocol. 1 μl of labelled cDNA was combined with 2 ul 50% glycerol/50% TE mixture, run on a microscope slide sized, 1.5% agarose gel (mould manufactured in-house) and scanned using a GeneTac LS IV scanner (Genomic Solutions, Huntingdon, Cambs. UK). Control and experimental samples were then combined and prepared for hybridization.

### Hybridization

Array slides were incubated in prehybridization buffer for 1 hour at 42°C (50% formamide, 5 × SSC, 0.1% SDS, 1% BSA). Targets were dried down via vacuum centrifugation then resuspended in 50 μl hybe solution (49.9% de-ionised formamide, 49.9% 20 × SSC, 0.2% SDS) with added 1 μl Cot1 DNA and 1 μl poly A oligo as blocking agents, heated to 95°C for 5 minutes and then added to the face of one slide. The printed face of the second slide of the pair was then placed face to face with the first, using the same probe. Slide pairs were then placed in a humidified container and incubated for 24–48 hours at 42°C. Following hybridization, slides were washed once in Wash solution 1 (1× SSC, 2% SDS, filtered autoclaved ddH2O) for 20 minutes, then twice in Wash solution 2 (0.1× SSC, 0.2% SDS, filtered autoclaved distilled deionised H2O (ddH2O) for 20 minutes each. Slides were dipped in nuclease free filtered water, then spray dried, finally, the backs of the slides were cleaned with filtered autoclaved ddH2O, then wiped with 100% EtOH, then wiped dry and scanned.

### Scanning

Scans were carried out at 12.5 μm, using the averaging setting (GeneTac LSIV scanner, (Genomic Solutions, Cambridgeshire, UK)). It is possible to carry out quick draft scans using this scanner. Gain and black settings, which affect image intensity and background, were varied slightly in order to optimize the signal/noise ratio for each channel and each slide before proper scans were initiated.

### MIAME standards

In adherence with MIAME standards [23], all data sets have been submitted to the GEO database  and are fully MIAME compliant. Undifferentiated ES cell comparison data are described in GSE8625, while EB differentiation comparisons are described in GSE8766. The results from these experiments were compared with an array comparison (described elsewhere) between undifferentiated ES colonies and microdissected inner cell masses from embryonic day 3.5, 4.5, and delayed blastocysts and microdissected day 5.5 and day 6.5 embryonic ectoderm (GSE8881, [24]).

### Array Platforms

Undifferentiated ES cell array experiments (GSE8625) were carried out using NIA 15K slides printed in the Cardiff Microarray Facility. These consisted of 17136 spots, printed in 12 × 4 mini grids, each with 17 rows and 21 columns and are described more fully in GEO, platform accession number GPL5530. EB differentiation comparisons (GSE8766) were carried out using NIA 15K slides printed by the HGMP. These consist of 2 slides, 17280 spots per slide, including control spots, empties and landmarks. 2 duplicate spots are printed per slide for each clone. These slides are described more fully in GEO, platform accession number GPL5735. The array slides used for GSE8881 were also printed in Cardiff and contain 16128 spots, printed in 12 × 4 mini grids, each with 16 rows and 21 columns. The GEO platform accession number for these is GPL5771. Differences in spot number relate to differences in the number of landmarks and other controls printed; the core NIA 15K set is present on each of these platforms

### Experimental design, Image analysis

Undifferentiated ES cell arrays were carried out using samples from undifferentiated IMT11, SMHBL6.3 and HM1 ES cells. EB arrays were carried out using samples from Day0 (undifferentiated), Day1, Day2, Day3 and Day 4 differentiated EBs, generated in the presence and absence of LIF (9 samples in total). Each experimental sample was hybridized on a slide with a pooled control derived from an equal amount of all experimental samples. ES arrays were repeated 12 times (this print run contained no duplicate spots). EB arrays were repeated twice. Duplicate spots were present on each array, giving four repeats total for each gene. Scanned images were stored and filtered, then analysed using ImaGene™ 5.5 (BioDiscovery). This series of array experiments was carried out using the NIA15K set [25]. ESTs comprising this set were isolated from a variety of embryonic stages and tissues. Arrays were hybridised with fluor switching, in order to counteract any issues of dye bias that may have arisen from direct labelling.

### Microarray Analysis

Array analyses for GEO entries GSE8625 and GSE8766 were carried out similarly to work described previously [26,27]. Output files from ImaGene were saved and analysed in MS Excel spreadsheet format. Each channel from each repetition was normalised via division by the mean intensity value. These data were collated and formatted for Significance Analysis of Microarrays (SAM; ). Genes that showed a fold change of 2 or above and were statistically significant above a delta value of 0.5 (which denotes an error rate of 5%) were selected for further appraisal. SAM, however, has a disadvantage; all replicates have to be in the same order, precluding any data filtration. Hence, we supplemented SAM analysis with a second analysis method as follows:

Following normalisation, we used approximately 700 blank spots per slide to calculate a mean background value + 2 standard deviations of that background value, for each channel. Genes that fell below this cut-off in BOTH control and experimental channels were removed, along with genes with a fold change of < 2. This filtered gene list was compared with that from SAM; genes appearing as differentially regulated using both methods were deemed significant (fold change > 2, above background + 2 SD in at least one channel, delta value of 0.5).

**Figure 1:**
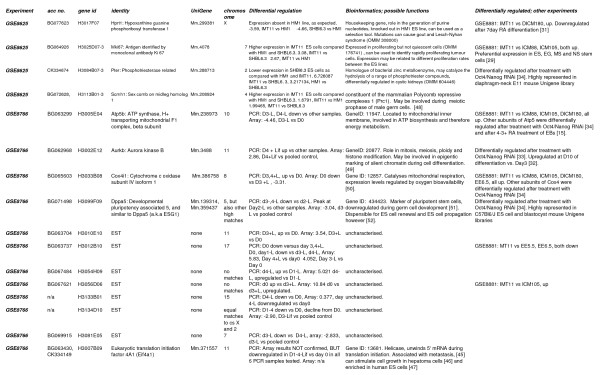
**Bioinformatic analysis of confirmed genes (part 1)**. From top left, columns are as follows: **Experiment**: this column indicates whether the gene confirmed is from the GSE8625 (undifferentiated ES cell lines) or GSE8766 (EB differentiation) gene lists. **Acc no**: GenBank accession number. **Gene ID**: the NIA clone ID, which is referred to in the NCBI nucleotide database as Gene ID. **Identity **refers to the official NCBI gene name to which the gene relates (where this is known). **UniGene**: refers to the UniGene cluster to which the EST has been assigned (where known). **Chromosome **indicates the mouse chromosome to which the EST maps. **Differential regulation **gives the nature of the expression change and the fold change derived from array analysis. Bioinformatics indicates possible functions related to differentiation obtained from searches of the NCBI database and the literature. **Differentially regulated:other experiments **describes if the EST/gene in question has been identified as differentially regulated in our own study of ES cells versus compartments of early embryos (GSE8881), and/or similar published work. Abbreviations associated with GSE8881: IMT11: undifferentiated IMT11 ES cells. ICM88 and ICM105 = blastocyst inner cell mass, 88 hours post coitum, 105 hours post coitum. DICM136, DICM180 = delayed blastocyst inner cell mass, 136 hours post coitum, 180 hours post coitum. EE5.5, EE6.5 = embryonic ectoderm, 5.5 and 6.5 days post coitum. For methods and more detailed descriptions of this experiment, see GEO, GSE8881, [24].

**Figure 2:**
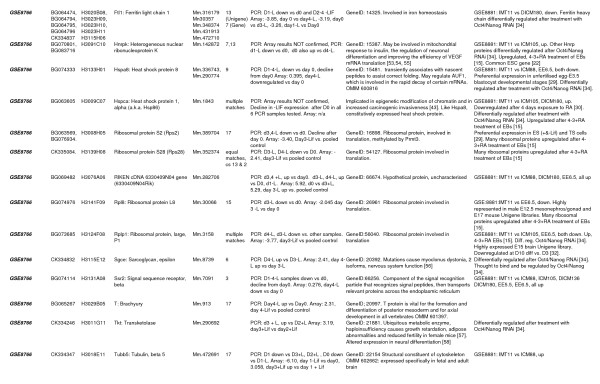
**Bioinformatic analysis of confirmed genes (part 2)**. From top left, columns are as follows: **Experiment**: this column indicates whether the gene confirmed is from the GSE8625 (undifferentiated ES cell lines) or GSE8766 (EB differentiation) gene lists. **Acc no**: GenBank accession number. **Gene ID**: the NIA clone ID, which is referred to in the NCBI nucleotide database as Gene ID. **Identity **refers to the official NCBI gene name to which the gene relates (where this is known). **UniGene**: refers to the UniGene cluster to which the EST has been assigned (where known). **Chromosome **indicates the mouse chromosome to which the EST maps. **Differential regulation **gives the nature of the expression change and the fold change derived from array analysis. Bioinformatics indicates possible functions related to differentiation obtained from searches of the NCBI database and the literature. **Differentially regulated:other experiments **describes if the EST/gene in question has been identified as differentially regulated in our own study of ES cells versus compartments of early embryos (GSE8881), and/or similar published work. Abbreviations associated with GSE8881: IMT11: undifferentiated IMT11 ES cells. ICM88 and ICM105 = blastocyst inner cell mass, 88 hours post coitum, 105 hours post coitum. DICM136, DICM180 = delayed blastocyst inner cell mass, 136 hours post coitum, 180 hours post coitum. EE5.5, EE6.5 = embryonic ectoderm, 5.5 and 6.5 days post coitum. For methods and more detailed descriptions of this experiment, see GEO, GSE8881, [24].

Samples were compared with pooled controls; sets of 4 experimental replicates were also compared with normalised samples from each of the other experimental samples. A "master list" of genes from all analyses was generated (see Figures 1 and 2 (all confirmed genes) and Additional File 1 (all unconfirmed genes)).

### Primer design

Significant ESTs were subjected to bioinformatic analysis via BLAST against the mouse genome, and, where available, against the reference sequence from the UniGene cluster to which they belonged. ESTs with sequences too short or too poor for primer design were rejected at this stage. Adequate EST sequences were used for primer design with Primer3 [28]), using sequence that matched the genome and/or RefSeq sequence. This analysis was carried out in order to rule out primer mismatches due to areas of erroneous first pass sequence. Primer sequences are given in Table 1.

**Table 1:** Primer sequences.

A) Marker/Housekeeping genes (note: Brachyury, Beta-actin, Hprt and Gapdh also came up in the arrays)		
Gene	Primer sequence	PCR fragment size (bp)

Oct4 F	GAGCACGAGTGGAAAGCAAC	521
Oct4 R	CGCCGGTTACAGAACCATAC	
NANOG F	TTACAAGGGTCTGCTACTGAGATG	431
NANOG R	GCAATGGATGCTGGGATACT	
**18Smm **F	GTAACCCGTTGAACCCCATT	150
**18Smm **R	CCATCCAATCGGTAGTAGCG	
GscF	CAGATGCTGCCCTACATGAAC	157
GscR	TCTGGGTACTTCGTCTCCTGG	
Fgf5 F	TGTGTCTCAGGGGATTGTAGG	136
Fgf5 R	AGCTGTTTTCTTGGAATCTCTCC	
KDR F	TTTGGCAAATACAACCCTTCAGA	112
KDR R	GCAGAAGATACTGTCACCACC	
Hprt F	CACGGACTAGAACACCTGC	229
Hprt R	GCTGGTGAAAAGGACCTCT	
Brachyury F	CATGTACTCTTTCTTGCTGG	312
Brachyury R	GGTCTCGGGAAAGCAGTGGC	
GATA4 F	CCCTACCCAGCCTACATGG	138
GATA4 R	ACATATCGAGATTGGGGTGTCT	
Rex1 F	CGTGTAACATACACCATCCG	128
Rex1 R	GAAATCCTCTTCCAGAATGG	
Nestin F	CCGCTTCCGCTGGGTCACTGT	227
Nestin R	CTGAGCAGCTGGTTCTGCTCCT	
NodalF	TTCAAGCCTGTTGGGCTCTAC	162
Nodal R	TCCGGTCACGTCCACATCTT	
GapdhF:	ACCACAGTCCATGCCATCAC	432
Gapdh R:	TCCACCACCCTGTTGCTGTA	
B-actinF:	CGTGGGCCGCCCTAGGCACCA	242
B-actin R:	TTGGCCTTAGGGTTCAGGGGG	
		
B) Primers, Confirmed and interesting genes:		

GSE8766		

Gene	Primer sequence	PCR fragment size (bp)

Aur F	AAATTGAAAGGAATCAGACTAGA	147
Aur R	GACCACTGTCTGTAACACCC	
Atp5b F	CCTGCATGGAAGGAAACCTG	238
Atp5b R	GTCACATGGGGAAGCTGGTG	
HnrpkF:	CCCCAACCCTGTTTGTAAGG	293
HnrpkR:	GGACCAGATACAGAACGCACA	
Dppa5F:	TCGGAGACACAAGGACTGGA	269
Dppa5R:	CCCACAGGGATCTCGAATGTC	
SgceF:	TGTCACGGTATTTGGTTCTCAA	170
SgceR:	CGCAGACTACAGGTAAATGGTA	
Rplp1F:	ACCGAAGCCCATGTCATCTT	211
Rplp1R:	CTTTCTGGCCTGGCTTGTTT	
Ssr2F:	TGGTTGAGTTCGGGGTAAGA	274
Ssr2R:	AGCGGGAGTTTGACAGGAGA	
**Hspa8F**:	GGGTTGCAGACTTTCTCCAGT	239
**Hspa8R**:	AAGGCTGAGGATGAGAAGCA	
Eif4a1 F:	CATCCAGCAGCGAGCTATTC	271
Eif4a1R:	CAGCTTCTGCACCTCAGCAC	
Rpl8 F:	CTCCAAAGGGATGCTCCACA	238
Rpl8R:	GCCACAGTCATCTCCCACAA	
Hspca1F:	ATCTGCACCAGCCTGCAAAG	176
Hspca1R:	AACTGGACTCGGGGAAGGAG	
BG063704 F:	GAACTCCAGACCTCCAGACCA	184
BG063704 R:	TTGCTTTGGGCAACAACTGA	
Rps2F:	TACCTGTTCTCCCTGCCCATT	180
Rps2R:	AACACCAAGACCAACGTGACC	
BG069915F:	GGAGTATGGAACGACCCTCTCA	201
BG069915R:	GAGCAGTGATTCTCAACCTTGC	
BG067484F:	GCCTCGATCAGAAGGACTTG	193
BG067484R:	GACCCGCTGAATTTAAGCAT	
BG067621F:	GCTCCCAAGATCCAACTACGA	257
BG067621R:	AGCCTGAGAAACGGCTACCA	
Cox4i1F:	CGCAGTGAAGCCAATGAAGA	246
Cox4i1R:	GCTTTCCCCACTTACGCTGA	
Ftl1F:	GCTGCCTAGTGGCTTGAGAGG	216
Ftl1R:	ATGGGCAACCATCTGACCAA	
TktF:	TATGGACTGGCCCTCGCTAA	286
TktR:	GGGAGCCACAGAGGTTGATG	
**BG063737F:**	GACGAGCACACAGGGAAACC	300
**BG063737R**;	GGAGAGAAGGAGGGGCAAGA	
BG069482F:	CCCTCGGATACCTGATGCTG	167
BG069482R:	TGAGAAATGACGGAGCCTTG	
Tubb5F:	TGGGAGGTGATAAGCGATGAA	257
Tubb5R:	GGCCTTTAGCCCAGTTGTTG	
Rps28 F:	CAGGTGCGAGTGGAATTCATG	198
Rps28 R:	TGCTTTATTTAACAGTTGCAGATCA	
H3133B01F:	CAGCCATTCAGCAAAGGAGA	283
H3133B01R:	TCTTGGGCAGGGTCTGTAGG	
H3134D10 F:	GCTCGGCTGTGTCAAGATGAAG	227
H3134D10 R:	CATGGGTCAGAACACCTTGCTT	
		
GSE8625:		

Mki67F:	CCTTGGCTTAGGTTCACTGTCC	250
Mki67R:	TGCAGAATCCAGATGATGGAGC	
PterF;	CATGTCCCACCTTGACAGGAC	245
PterR;	CCGTACTTCATCAACCGATGC	
Scmh1F:	GGACCCAGTGTAGGAAGAGAGACC	206
Scmh1R:	ATTGCTTCTGGCGTTTGGAC	

Primers in bold were also used for Q-PCR.

### Bioinformatic analysis

Bioinformatic analysis of confirmed genes was also carried out in order to identify putative function in differentiation. Accession numbers were used to comprehensively search the NCBI databases . Data compiled from various NCBI databases (UniGene, Homologene, OMIM, LocusLink, PubMed etc.) are shown in Figures 1 and 2. Selected marker genes were chosen on the basis of a known role in maintenance of pluripotency, or early development, or because they are well known housekeeping genes. Primers were designed for these from the appropriate reference sequence, using Primer3 as above. Datasets derived from ES cell differentiation subtractive EST library studies [15,29] or microarrays [30-38] were also searched for the presence of the genes we confirmed in this study (Figures 1 and 2).

### RT PCR array confirmations

We used semi-quantitative RT-PCR to confirm differentially regulated genes. As most significant genes appeared to be differentially regulated at various different stages of differentiation, we tested all genes against Day 0, and Day1–4 EBs, + and - LIF. Three biological replicates of 1000 cell Day 1–4 EBs and 3 replicates of 750 cell EBs (+ and - LIF) were used for confirmations, including No RT controls. In order to be deemed confirmed, we asked that a gene demonstrate a consistent expression pattern in at least 4 out of 6 PCR tests. The number of cycles required for minimum visibility was identified and PCRs were optimised at the Tm indicated for each primer pair, such that only single bands appeared. PCR bands obtained for housekeeping controls were of even intensity at minimum visibility cycles before testing other genes. PCRs were carried out in 20 μl volumes using 0.025 μmol of each dNTP (Invitrogen) and 1 unit Taq DNA polymerase (Sigma) per reaction. PCRs were usually carried out using PCR buffer (Sigma) containing 15 mM MgCl_2 _although magnesium concentrations, along with annealing temperature and cycle number, were varied where necessary in order to optimise results. Final PCR conditions are indicated for each gene in the appropriate figures (see Results). Primer sequences are given for all genes tested in Table 1 and Additional File 1. In order to confirm that semi-quantitative PCR was indeed representative of expression pattern changes, we studied the expression patterns of two confirmed genes, Hspa8 and BG063737, by quantitative PCR. Expression pattern changes achieved by q-PCR were very similar to the methods described above. Methods and results are given in Additional File 2[39].

## Results

### Undifferentiated ES cell arrays

**Figure 3:**
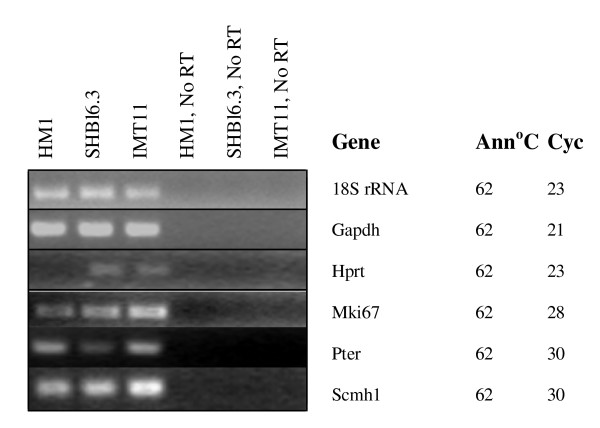
**Genes confirmed as differentially regulated between the 3 different undifferentiated ES cell lines, HM1, SHBl6.3 and IMT11**. Ann = PCR annealing temperature, Cyc = number of PCR cycles used. Beta-actin was not used as a housekeeping control as the arrays noted it as variable. It was variable, but the variation in expression patterns between repeats was very high, such that array results for this gene could not be confirmed. 4 genes were confirmed; Mki67 and Scmh1 showed highest expression in IMT11 cells, Pter showed lower expression in SHBl6.3 cells than in the other two lines, while Hprt expression was absent from HM1 cells as expected.

We studied expression differences between 3 ES cell lines, IMT11 (derived from 129 mice), HM1 (which is Hprt negative) and SMHBl6 (derived from C57Bl6/J mice) (GEO, GSE8625). 21 genes, including Hprt, were identified from the arrays as possibly differentially regulated, four were confirmed by RT-PCR; Hprt, Mki67, Pter and Scmh1 (see Figure 3). Hprt was not expressed in HM1 cells, as expected. Mki67 and Scmh1 were upregulated in IMT11 cells, while Pter was downregulated in SHBl6.3 cells.

### Optimisation of embryoid body differentiation

**Figure 4:**
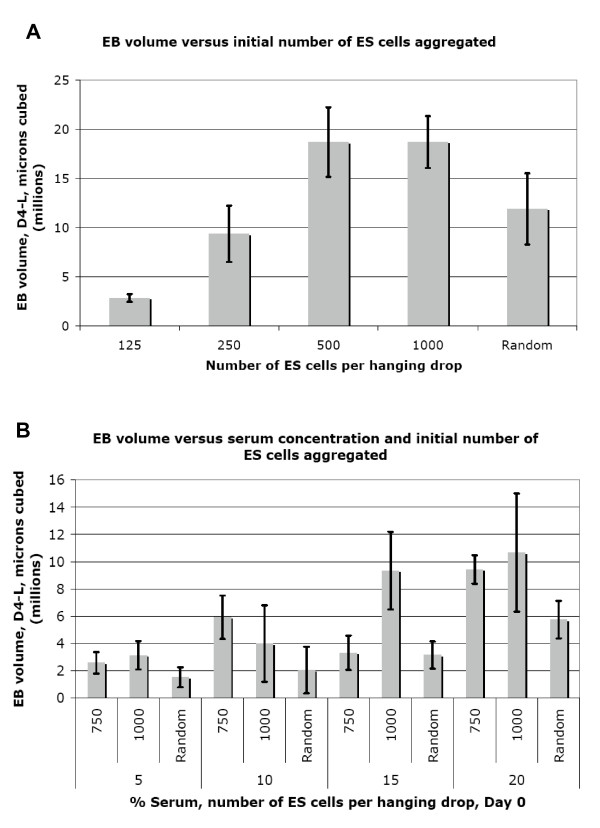
**(a) Diameter of EBs at Day 4-LIF plotted against the initial number of cells aggregated via hanging drop on Day 0 b) Diameter of EBs at Day 4-LIF plotted against the serum concentration in the differentiation medium and initial number of cells aggregated via hanging drop on Day 0**.

IMT11 ES cells were used to generate EBs of 125, 250, 500, 750 and 1000 cells via hanging drops, using ES medium - LIF and 20% serum (FBS + NBS). An upper limit of 1000 cells was chosen as we often observed substantial interior necrosis and RNA degradation in larger EBs by Day 4 of differentiation. Analysis of EB size measured at day 4 shows little variation in diameter between EBs initiated with a cell number of 500 or greater; size constraints may begin to apply once a certain diameter is reached (Figures 4a + b). RT-PCR showed that 125–500 cell EBs failed to show expression of genes such as *brachyury *by Day 4. Furthermore, the amounts of RNA retrieved were insufficient for array analysis at Days 1 and 2 (results not shown). 750 and 1000 cell EBs showed expression of markers of all 3 germ layers (see below), and gave a better RNA yield at days 1 and 2.

**Figure 5:**
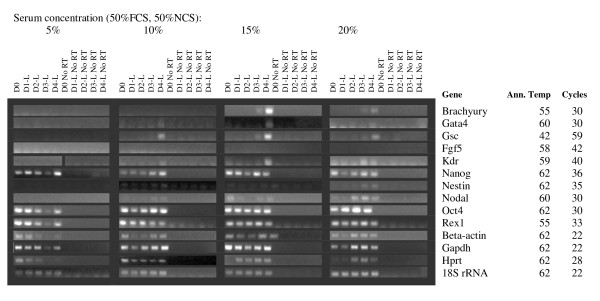
**RT-PCR of key marker genes (involved in maintenance of pluripotency or early development) and housekeeping controls at varying medium serum concentrations**. Starting size 750 cells. **Marker Genes:** Oct4, Rex1 and Nanog are markers of ES cell pluripotency, Beta-actin, Gapdh, Hprt and 18S rRNA are commonly used housekeeping genes; however, we have found that the first 3 vary unpredictably in expression during differentiation. Brachyury and nestin are markers of early mesodermal and neurectodermal differentiation respectively; Nestin is considered a CNS stem cell marker. Goosecoid is a marker of the Spemann organizer and gastrulation. Gata 4 is expressed in yolk sac endoderm and during heart formation. Nodal is expressed during gastrulation and is involved in anterior-posterior and visceral endodermal patterning. Kdr (a.k.a. Flk1, VegfR) is exclusively expressed in endothelial cells and defines multipotent haematopoietic stem cells. Fgf 5 is a marker of primitive ectoderm.

**Figure 6:**
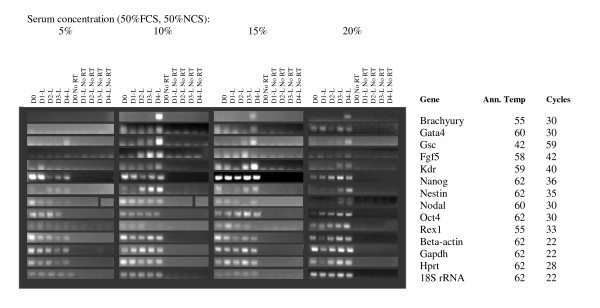
**RT-PCR of key marker genes (involved in maintenance of pluripotency or early development) and housekeeping controls at varying medium serum concentrations**. Starting size 1000 cells. **Marker Genes:** Oct4, Rex1 and Nanog are markers of ES cell pluripotency, Beta-actin, Gapdh, Hprt and 18S rRNA are commonly used housekeeping genes; however, we have found that the first 3 vary unpredictably in expression during differentiation. Brachyury and nestin are markers of early mesodermal and neurectodermal differentiation respectively; Nestin is considered a CNS stem cell marker. Goosecoid is a marker of the Spemann organizer and gastrulation. Gata 4 is expressed in yolk sac endoderm and during heart formation. Nodal is expressed during gastrulation and is involved in anterior-posterior and visceral endodermal patterning. Kdr (a.k.a. Flk1, VegfR) is exclusively expressed in endothelial cells and defines multipotent haematopoietic stem cells. Fgf 5 is a marker of primitive ectoderm.

**Figure 7:**
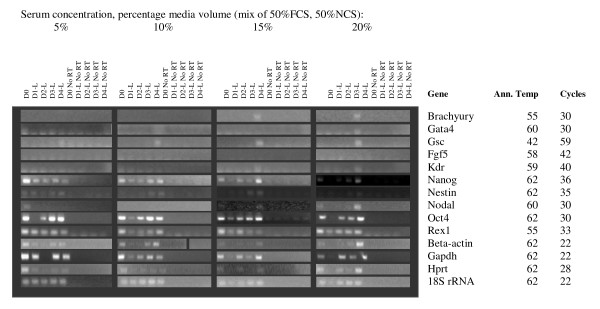
**RT-PCR of key marker genes (involved in maintenance of pluripotency or early development) and housekeeping controls at varying medium serum concentrations**. EBs formed via random aggregation. **Marker Genes:** Oct4, Rex1 and Nanog are markers of ES cell pluripotency, Beta-actin, Gapdh, Hprt and 18S rRNA are commonly used housekeeping genes; however, we have found that the first 3 vary unpredictably in expression during differentiation. Brachyury and nestin are markers of early mesodermal and neurectodermal differentiation respectively; Nestin is considered a CNS stem cell marker. Goosecoid is a marker of the Spemann organizer and gastrulation. Gata 4 is expressed in yolk sac endoderm and during heart formation. Nodal is expressed during gastrulation and is involved in anterior-posterior and visceral endodermal patterning. Kdr (a.k.a. Flk1, VegfR) is exclusively expressed in endothelial cells and defines multipotent haematopoietic stem cells. Fgf 5 is a marker of primitive ectoderm.

Higher serum concentrations are known to promote maintenance of pluripotency in cultures of undifferentiated ES cells, and also provide more nutrients and pH stability to the medium. We generated random, 750 cell and 1000 cell EBs in ES media without LIF, containing 0, 5, 10, 15 and 20% serum (1:1 mix of FBS + NBS). EBs cultured with no serum were dead after 24 hours and are therefore not shown in data analysis. Measurements of EB diameter showed that 20% serum promoted the largest EBs at Day 4 for 750 cell and randomly generated EBs, while 1000 cell EBs reached similar sizes with 15% and 20% serum concentrations (Figure 4b). RNA was extracted at days 1–4 of differentiation and analysed by RT PCR. Results are shown in Figures 5, 6 and 7. The greatest range of gene expression was noted on days 3–4 in concentrations of 15–20% serum (Figures 5, 6 and 7). Moreover, Fgf5 was only expressed in EBs with a starting size of 1000C. One should note that the higher serum concentrations also promote the maintenance within the EB of undifferentiated cells; the expressions of markers of pluripotency such as Oct4, Nanog and Rex1 diminish at Day 4 in 5% serum, but maintain and even increase expression levels at 15–20% serum. Given more varied gene expression profiles (suggesting that a greater range of cell types could be obtained eventually from such a protocol over time), size and greater RNA yield, we decided to use 1000 cell EBs grown in 20% serum for our subsequent array experiments.

### Array analysis

Array results from Day 1–4 EBs, grown in both the presence and the absence of LIF, were compared at all stages with the pooled control (equal amounts of Day 0–4 ES cells + EBs, both with and without LIF), and also with each other, when analysing the arrays. 128 ESTs selected for follow-up were differentially regulated in all 4 repeats and were statistically significant according to SAM. Many ESTs were differentially regulated in more than one comparison. Removal of redundancy (more than one EST mapping to different parts of the same gene) and elimination of those ESTs where sequence quality was insufficient for primer design resulted in a master list of 104 genes. Notably, beta-actin and Gapdh were differentially regulated, so 18S rRNA was used as a housekeeping gene instead.

**Figure 8:**
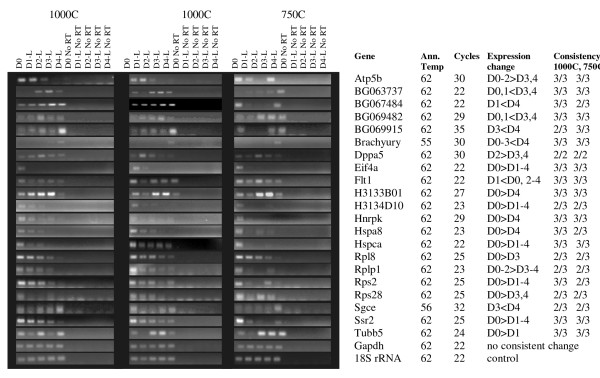
**Confirmed genes, up- or down-regulated during differentiation without LIF**. Ann temp = PCR annealing temperature, Cycles = number of PCR cycles used. Expression change marks the direction of expression changes as shown by RT-PCR. Consistency 1000C, 750C refers to the number of replicates out of three that show the same change (always at least 2), this information is given as space constraints prevent showing all.

**Figure 9:**
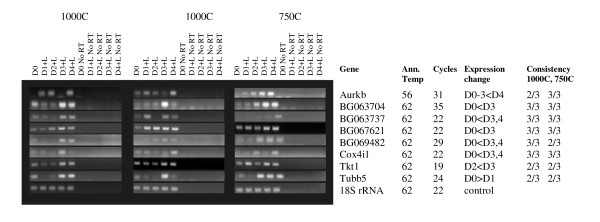
**Confirmed genes, up- or down-regulated during differentiation in the presence of LIF.** Ann temp = PCR annealing temperature, Cycles = number of PCR cycles used. Expression change marks the direction of expression changes as shown by RT-PCR. Consistency 1000C, 750C refers to the number of replicates out of three that show the same change (always at least 2), this information is given as space constraints prevent showing all.

Given problems with reproducibility that have been noted with stem cell arrays [20-22], RT-PCRs were carried out on 3 sets each of 1000 cell and 750 cell EB samples; differentially regulated genes showed a reproducible expression pattern change in at least 4/6 samples. 18 genes were differentially regulated in -LIF samples; a further 8 were differentially regulated in + LIF samples. 3 genes appeared in both datasets (Tubb5 and 2 uncharacterised genes; BG063737 and BG069482, see Figures 8 and 9 and Figures 1 and 2). None of these 23 genes appeared in the list of 3 that were differentially regulated between different undifferentiated ES cell lines (GSE8625). A further 3 genes did not show the exact expression patterns predicted by the array, but were dramatically downregulated on induction of differentiation and are therefore also presented in Figure 8. This gave a total of 26 genes we deemed differentially regulated during ES cell differentiation. Q-PCR analysis of two confirmed genes, Hspa8 and BG063737, gave very similar results to semi-quantitative PCR (Additional File 2).

We then compared this dataset with that from another of our array experiments, which compared undifferentiated IMT11 ES cells with embryonic inner cell masses (the tissue from which ES cells are derived) and day 5.5 and day 6.5 embryonic ectoderm (see Figures 1 and 2, GSE8881, methods described [24]), Thirteen of the above 26 genes were also up- or down-regulated in this study, indicating their importance in early development and differentiation. Fourteen of the known genes were also noted in other array or subtractive EST library studies using material from different ES cell differentiation experiments (see Figures 1 and 2). Interestingly, two of the four genes that varied between undifferentiated cell lines were also identified by this study.

### Amplification from single EBs

**Figure 10:**
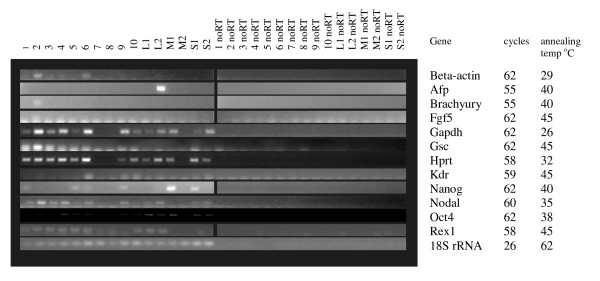
**Expression of differentiation markers in EB differentiation**. EBs were allowed to differentiate in standard ES medium -LIF for 4 days. To determine whether each EB was consistently expressing marker genes or if these genes were only expressed in a minority of EBs, we used an RNA amplification kit to carry out one round of amplification on RNA from 16 individual EBs harvested on day 4 of differentiation, -LIF. Ten (1–10) were derived from 1000 cell hanging drops and were of similar sizes. We also harvested 6 EBs which had been allowed to aggregate randomly in solution, these were varied in size: 2 large (L1+L2), 2 medium (M1+M2) and 2 small (S1+S2). Medium EBs were the same size as those obtained from the hanging drops.

Our arrays have successfully identified 23 genes that show expression changes during EB differentiation (plus an additional 3 that did not show the exact expression changes indicated by the array). However, 23 genes represents a low confirmation rate (22%) given the initial 104 genes tested. The consistency of gene expression in individual embryoid bodies was therefore tested in order to assess how this variation might influence the reproducibility of ES differentiation protocols that are based on EBs. We studied 10 individual 1000 cell Day 4 EBs from the same tissue culture dish and 2 small, 2 medium and 2 large EBs from a similar plate of Day 4-L randomly aggregated EBs (medium represented the same size as those derived from the hanging drop method, large EBs were roughly double this size, while small ones were half this size). 18S rRNA was used as a housekeeping gene control. Expression of Gapdh and Hprt was noted in most EBs, albeit at varying levels. Expression of Nodal and Rex1 was also noted in a majority of samples. Nanog, Oct4, Goosecoid and beta-actin were expressed in 50% or less of EBs, while Afp, Kdr, Brachyury and Fgf5 were expressed in 1–3 EBs out of 16 only (Figure 10).

## Discussion

We have shown that undifferentiated mouse ES cells, regardless of strain of origin, or absence of Hprt, maintain a very invariant expression pattern under identical culture conditions. Given genetic variation between mouse strains of origin (129Sv/Ev and C57Bl6/J) and absence of the Hprt gene in one line, this result is perhaps surprising, but would imply that the culture conditions that maintain pluripotency may be quite restrictive in terms of gene expression pattern. Hprt was not expressed in HM1 cells as expected. The relevance of Pter differential regulation is unclear, unless this gene is functionally associated with the Hprt pathway. Mki67 is a marker of proliferating cells; its upregulation in IMT11 cells might suggest a higher growth rate, although this has not been tested. Scmh1 may be involved in the meiotic prophase of male germ cells; ES cells are similar to germ cells, but the significance of the differential expression is unclear. We should note that, apart from HM1, expression differences noted were minor. We selected IMT11 cells for further study.

EB growth optimisation shows that EBs of 500–1000 cells reach a size plateau at 20% serum, suggesting that size constraints apply to growth once a certain size is reached. This may be related to the decreased rate of gas and nutrient diffusion to cells in the centres, but EBs are thought to be loosely packed and should therefore not suffer from a buildup of toxic waste products [40,41] EBs created using cell numbers of 1–1000 were previously found to attain a maximum of approximately 30,000 cells on Day 12 of differentiation, regardless of culture methods used or initial cell number [41]. Alternatively, growth constraints may arise from the absence of some necessary developmental cue, signalling during cavitation or from decreased cell division rates upon initiation of differentiation, which may happen sooner with a larger initial size [41,42].

Variation of initial size and serum concentration also influences gene expression patterns after 4 days of differentiation. We selected an initial size of 1000 cells and a serum concentration of 20% for our array experiments, on the grounds that these conditions were optimal for growth (and therefore RNA yield), gave the widest range of marker gene expression (implying a greater range of differentiated progeny) and were likely to bear more similarity to early embryonic differentiation.

Microarrays have identified 23 genes, which are differentially regulated during ES cell differentiation; a further 3, while not demonstrating the expression patterns predicted by the array, showed dramatic down-regulation upon induction of differentiation without LIF and are therefore discussed here. Detailed synopses of functional information available is given in Figure 1; a number of functional studies are also of interest [43-59].

13 of these genes are implicated in the early development of pre- and post- implantation embryos, while 14 are highlighted by other studies and/or preferentially expressed in embryonic EST libraries. Given the variation in ES cell lines, differentiation protocols and timepoints studied by others, this overlap is reasonably significant [15,29-38]. The functions of 7 ESTs and 1 hypothetical protein are presently uncharacterised.

The remaining genes fall into a number of different classes. Hspca (a.k.a Hspc1, Hsp89a, Hsp90a) and Hspa8 are immediately downregulated on induction of differentiation. Both are constitutively expressed heatshock proteins, a class of proteins commonly upregulated in response to cellular stress (OMIM 140571) and also implicated in ES differentiation [43]. Heatshock proteins can function as chaperones; ie, they assist in the correct folding of nascent proteins. Ssr2, another protein downregulated upon onset of differentiation, is also associated with nascent polypeptides, although it forms part of a complex that recognises signal peptides, resulting in transport of the relevant protein across the endoplasmic reticulum. Hspca has been implicated in epigenetic modifications of chromatin (disruption during *Drosophila *development results in heritable morphogenic alterations) and in increased carcinogenic invasiveness [44]. Pluripotent cells, such as ES cells, demonstrate uniquely dynamic chromatin, "breathing chromatin", which allows availability of a large proportion of the genome for immediate transcriptional activity [45]. Perhaps reductions in Hspca expression during differentiation may result in changes to chromatin structure that result in the tighter histone binding characteristic of differentiated cells and their committed precursors.

Notably, a related class of 5 genes also shows downregulation upon initiation of differentiation; Eif4a1, Rplp1, Rpl8, Rps2 and Rps28. Eif4a1 encodes a translation initiation factor that could be involved in selective regulation of protein expression, while the remaining four genes are structural constituent of the ribosome. Expression of Eif4a1 is associated with increased metastasis in certain cancers [46], can stimulate cell growth in hepatoma cells [47] and is enriched in populations of human ES cells [48]. We have previously noted the upregulation of similar genes involved in translation in response to neural differentiation of ES cells [15]; however, this upregulation was noted after 7 days of EB differentiation, following the addition of retinoic acid on Day 4. It is possible that ribosomal constituents may play a role in complex changes that occur in gene expression in response to changing signals at different stages of differentiation.

A third class of genes comprises those that are involved in energy metabolism and other ubiquitous metabolic processes (Atp5b, Cox4i1, Ftl1, transketolase, Hnrpk). Energy and metabolic requirements may change during differentiation; alternatively, these genes may have alternative functions in these processes. Perhaps surprisingly, only 3 genes, Brachyury, Aurkb and Dppa5 have identified roles in the maintenance of pluripotency, epigenetic remodelling and early development (see Figure 1); we would have expected this class of gene to be better represented. However, larger EBs maintain a core of pluripotent cells that maintain LIF expression and also, therefore, continue to express genes such as Oct4, Nanog and Rex1 for some time. This is obvious from our PCRs of these marker genes; expression can dip a little on Day 1 but normally resumes by Day3–4 of differentiation. Other genes, such as Laminin, are known to display differential expression during early development; laminin was among the 104 genes selected for PCR confirmation. However, expression varied so much between PCR replicates that there was no consistent pattern of variation. This finding was common to a majority of the genes tested; this would not rule out their importance to early developmental processes, however, it would suggest that those genes that did show consistent expression changes may indeed be very important to early development; we were stringent in our selection of differentially regulated genes.

The identification of 26 genes which alter in expression pattern in ES cell differentiation will aid the understanding of early development and *in vitro *differentiation. However, there are important implications of the low confirmation rates noted in this paper, and of the variability in marker gene expression profiles between individual EBs of homogeneous cell number and size, which originated from two tissue culture dishes. Firstly, ES differentiation protocols using EBs as a starting point may always generate very variable results, despite attempts to standardize them such as using a uniform EB starting size. This would necessarily limit their therapeutic use; development of differentiation protocols that yield more uniform populations of progenitors without the use of EBs may be preferable (such as the majority of current neural differentiation methods)[17,18]. On the other hand, the ability of EBs to generate a wide variety of precursors that could later be selected for subtypes of choice may be an advantage in certain circumstances. Secondly, this finding would imply that the lack of directional orientation in EBs as compared to early embryos is a source of chaotic variability.

## Conclusion

Stem cells provide, potentially, an unparalleled opportunity for treatment of any number of degenerative conditions. A deeper knowledge of stem cell differentiation and of signalling pathways activated therein, will increase our ability to direct the differentiation of stem cells *in vitro*. This study has advanced our knowledge of early ES cell differentiation in several key respects. Firstly, we have demonstrated that regardless of underlying genetic variation, the constraints of ES cell pluripotency seem to maintain a relatively invariant gene expression profile. Secondly, we have developed optimised tissue culture conditions that allow the widest range of differentiation potential. Thirdly, we have identified genes that are implicated by this study and others in the complex processes of early development; furthermore, given the number of biological repetitions and the EB size variation used when confirming these genes, we would regard our results as robust. Perhaps most importantly, when investigating low confirmation rates, we have shown that gene expression patterns of individual EBs vary markedly from each other, even when grown from the same number of starting cells, in the same culture. This implies that differentiation protocols involving EBs may always yield varying proportions of different cell types, no matter how rigorously conditions are controlled between different experimental replicates, and may suggest that a move away from EB based differentiations may be warranted, where possible.

## Authors' contributions

FCM carried out array analysis (GSE8625, GSE8766), bioinformatics, primer design, marker gene PCRs, most protocol optimisations, PCR confirmations (GSE8625), GEO submissions, individual embryoid body amplifications and PCR, manuscript preparation, submission and revisions. CSD provided EB RNA samples for PCR, most PCR confirmations (GSE8766) and q-PCR. ALH completed all array hybridizations, array experimental design and image analysis leading to provision of raw array data. MAW carried out image and statistical analysis of EB diameters, preparation of Figure 3, manuscript editing, figure compilation and assistance with bioinformatics. SMH carried out all experimental + some analytical work connected to GSE8881, as well as study conception and design, provision of logistical support and funding, supervision of participants, assistance with analysis and interpretation, tissue culture protocol optimization and manuscript editing. MJE was responsible for study conception and design, GSE8881 array analysis, logistical support and funding, supervision of participants, assistance with interpretation, manuscript editing. All authors have read the manuscript

## Supplementary Material

Additional file 1 **This table lists names and primer sequences of genes that were identified by both array experiments, but not confirmed**. Click here for file

Additional file 2 **Q-PCR confirmation of expression patterns of Hspa8 and BG063737 and comparison with semi-quantitative PCR results**. Q-PCR confirmation of expression patterns of Hspa8 and BG063737 and comparison with semi-quantitative PCR results. Q-PCR was carried out using an MJ-Research Peltier Thermal Cycler PTC-200 PCR machine and results were analysed using MJ Opticon Monitor 3.1.32 software using previously described methods [39]. Reaction volumes were 25 μl, comprised of 5 μl cDNA, 12.5 μλ PCR mix from the DyNAmo HS SYBR Green qPCR kit (Finnzymes) and 7.5 μl primer mix (25 picomolar). Cycles were as follows: 95°C for 15 mins followed by 34 cycles of 95°C for 30 s, 62°C for 30 s and 72°C for 30 s. Primer sequences are given in Table 1. In order to compare results with those obtained from semi-quantitative PCR, we used Scion Image (Scion Corporation) to measure relative band intensities for 3 repetitions, and processed the figures with reference to the Day 0 sample and the 18S housekeeping gene, such that they were in a similar format to the figures obtained by Q-PCR. While the figures obtained are not identical, the expression patterns seen in terms of trends of differential regulation are very similar; Hspa8 declines in expression from Day 0 while BG063737 dips at day 1-L and peaks at Day 3-L. Click here for file
