# Supplementary material for: Gene expression profiles during early differentiation of mouse embryonic stem cells
Source: BMC Dev Biol. 2009 Jan 9;9:5. doi: 10.1186/1471-213X-9-5 (PMC2656490; doi:10.1186/1471-213X-9-5)
Supplement: Additional file 1 — This table lists names and primer sequences of genes that were identified by both array experiments, but not confirmed. [file 1471-213X-9-5-S1.doc]

Supplementary file 1: Unconfirmed genes, primer sequences

a) ES undifferentiated, GSE8625:

CK334837F: CGGGCCTCCTACACCTACCT

CK334837R: CATGGCCTCCTGGGTTTTAC

BG063596F: TGTTCTTTCTCAACCCAATTTCC

BG063596R: TCGAGGCAAATGCACAGAAG

Cct7F: ATGGCACCACCTCAGTGACC

Cct7R: CCTTCTGCTGGGAGATCAGC

Ube2L3F: TGGCAAGGGCTTATTGTTCC

Ube2L3R: CAGTCTTGGTGGCTGGCTTC

Npc1F: TGAATGCGGTCTCCTTGGTC

Npc1R: CAAAGGCTAACACCACGATCC

Amd1F: GCACTGGCCCTCCAAATAGC

Amd1R: TTCTTGGTAGTTTCTGGACTGATGC

Dpp4F: CTTTCCCATCACCCTTGCTGT

Dpp4R: GGTCCAAGCATACAAACCAACC

BG063478F: AAACCAGCACTAGAAAAGTAGATCCT

BG063478R: TGTTTCCCCAGCATGTGTACC

BG063579F: CTTCCTGTGGGAGGCTCAAG

BG063579R: GAAGCTAAGGAGTTCCCATCCTG

Ddx3xF: GTGAGGGCTGGTCATTGCTG

Ddx3xR: AGCACTGTTCTCAAAGTTAATGCAAG

Tpm3F: GGCCGAGAAGAAACCACCTG

Tpm3R: CATCTCATCGCCCTCTCCTG

PicalmF: ACAGAAAGCACCGATCAACAG

PicalmR: AGGGCCTTTCCAAATGAATG

HspcbF: CATGAGCAGAGAGCCAGGTG

HspcbR: GCATCTACCGCATGATTAAACTAGG

Recc1F: GCTACAAAAGATGCCCACTGC

Recc1R: CCTCACCCTTTGCCAGAGTAAC

Smarca5F: TCCTGCTTCAGGGTCTCCAG

Smarc5R: CAGCAGCCACAAGATAATGTCAG

b) EB differentiation : GSE8766

Ak4F: GATACAATCACCCCACTTTTCCA

Ak4R: CGTCTGCTTGCTCCCTTCTC

BG077167F: AGGCCCTGACCACTCTGGAA

BG077167R: ACAAAGCGCCGGTTGTACTG

CK334244F: GGATTGTCACACAGCCCACA

CK334244R: GCCTCATGTCACGAACAGCA

BG063732 F: GCCTGCGTTGGATGCAATTC

BG063732 R: CGGAGCTGTGATGAACTGTGC

CK334258F: CGAAGTGGCGGAAGAAGAGA

CK334258R; TTACCGATGGCAGCACACAG

CK334282F: GCGTCTGGGTAATGACTTCCA

CK334282R: GGTTAGAGAGACTGCCAAAGTCCA

Rbx1F: TGCATCTCTCGATGGCTCAA

Rbx1R: CGGGAACACCAAACTCCAAA

Chchd2F: GATCGGTGAGACTTCGAGCA

Chchd2R: GTAGCCATCTGGGCCATCAG

Aldoa1 F: ctgccagcagaatggcattg

Aldoa1 R: ggctcgaccataggagaaag

Dnajc7F: TGCCTGCTTCCCTGTCTACC

Dnajc7R: AGGCATTCTTCGGTGGTCCT

Catna1F: CTATGCAGTCATCAGGCTTCTTTG

Catna1R: GCTTTATTCGGGCTCGTGTG

Rps15 F: gatctccacctggttgaagg

Rps15 R: tcgaccaactgctcgacatg

Rpl32F: GCGTTGGGATTGGTGACTCT

Rpl32R: TAAGCGAAACTGGCGGAAAC

BG068918F: AACACCAGATGAAGGGTGGA

BG068918R: CCTTACGGGGAAAACCATCA

BG069449F: AATGCCACGTTTGCCTATGA

BG069449R: CCCCTGCTGAAATCCCTACA

BG069712F: GCTCCCAAGATCCAACTACGA

BG069712R: AGCCTGAGAAACGGCTACCA

Rpl6F CAGCCTTTCGCTGCTCTGTAA

Rpl6R: ACAGTGGCTTGCTGCTTGTG

Arfrp2F:CCCAAGGCAGACACATTTCC

Arfrp2R:CAGACCAGGCATCTCCATCA

Bzw1F: AAAGTCTCTGGTGGCATTATTCTC

Bzw1R: GCTGCCTTCGTTGCTTTTCT

BG062966 F: GATTCTTCAGCTGACTGCAAGA

BG062966 R: CGATCGCCTGGAGAAATGAC

Rpl37 F: AAGGGAACGTCATCCTTTGGT

Rpl37R: AGACAATCTTTAGGTGCCTCATCC

BG063010 F: TCCATCTTCCACCCTACAGGAC

BG063010R: GATGCAACACACAGGCTCCA

Rps27L F: CCTGGCTTTTCCTCCTGTGG

Rps27L R: CCATGCCCCTGGCTAGAGAT

BG063232 F: AGGTGTTGGGCCTCTTGGTG

BG063232 R: CTCCATCCAGATCATGAAGG

H3004G04 F: ACACAGCTCATTTCATTGGTG

H3004G04 R: AATAGATTTGGTGCTCTACACTTACA

Trim28 F: ACCACAAGAGCCCCAGCTTC

Trim28 R: CCCCAGGAGTTTGCTCAAGA

Peg10F: GAAGTTCACCACGAAGAGTCAGTT

Peg10R: TCAGATGCGTGTTCCCTCTC

Rpl18F: TGTTGACATTCGCCACAACA

Rpl18R: TCCAGAATCCGCACATCATC

Rps18F: TTCCAGCACATTTTGCGAGT

Rps18R: CCTGGCTGTACTTCCCATCC

CK334683F: TAACATGCTGCGGATTGTGG

CK334683R: AACTTGAAGGGCCACAGGAA

H3099B07F: GCAGCCACTGATTGCACAGA

H3099B07R: CGTAGTGGTGGTGGTGGTGA

BG084332F: GCTGGCTAGGGTAACCAAAGTG

BG084332R: AACGCAACCTTCGAGCTTCT

H3099H01F: TCTGTGTAACCCTGACTGTCCT

H3099H01R: CCAGCTAGAAGCCAACCATT

BG071626F: GGGTGGTCCAGGGTTTCTTAC

BG071626R: AAGGGCATTCTCGGCTACAC

Klhl18F: TGGTGAAGCCCAGAGAGAGA

Klhl18R: TGAGCACACAAGCGAACAGA

Rps21F: CAAATTCCCGATTTCTTCTGCT

Rps21R: GCCGAGGTTGATAGGACCAC

Rpl3F: CTTCTTGGTCCCCACCACAC

Rpl3R: GGGCAGAAAGGCTACCATCA

Rabgef1F: GGCCTCCTACGGGTTTTCTT

Rabgef1R: TTTACGAGCAGTCGGTGGATT

Lamr1F: AGCCTGCTCCTCCTTCTCAA

Lamr1R: TTATGTCAACCTGCCCACCA

BG071626F: GGGTGGTCCAGGGTTTCTTAC

BG071626R: AAGGGCATTCTCGGCTACAC

Rpl10aF: GGCAGCAGTGAGGTTTATTGG

Rpl10aR: GCCAAAGTGGATGAGGTGAA

Rpl10F: GACACCACAGCCATCAGGAA

Rpl10R: TCATGTCCATCCGAACCAAG

H3125E05F: AGGCTGAAGCGCAAGAGAAG

H3125E05R: GGCAGAGGGACTGTTTTGGT

CK335084 F: CAGGTGCGAGTGGAATTCATG

CK335084 R: TGCTTTATTTAACAGTTGCAGATCA

Hn1 F: TCCAGGGAGCTTGCTTGTGT

Hn1R: TTCCTGTGCCATGTGGAGTG

BG075587F: GGCAAGGGTTCAAGGTTATCTG

BG075587R: TCCTGTCTCAGCCTCCCAAG

Rps20F: TTAGGCATCTGCAATGGTGA

Rps20R: ACCTTGTGGTGAAGGTTCCA

Pcbp2F: TGCAAACCCAGTGGAAGGAT

Pcbp2R: TCATGGGTGGTGAACAGCAG

Dnclc1F: TCACTGGGTGTTTGGCACAG

Dnclc1R: TTGCGGCCCATATCAAGAAG

Cox8aF; TGATTGCAGAAGAGGTGACTGG

Cox8aR: CATTGGGCTCACTTCCTGCT

H3010A09F: GCAGGCCATCAAACAGGAGT

H3010A09R: GGCTGGTCTCCCTCATCACC

Hist1h4mF: GTGCTCCGTGTAGGTGACG

Hist1h4mR: ATAACATCCAGGGCATCACG

CalrF: ACCAGATGAGGGCTGAAGGA

CalrR: TGGCTGCTCCCAATAATGTC

Eno1F:CCTCCCATGTCACTGCTTCC

Eno1R: TGTTGGACTTCTGCGGCTTT

ArbpF: CGAAGAGACCGAATCCCATA

ArbpR: AAAAGGTCAAGGCCTTCCTG

Eif3s6ipF: GCGTCTGGCAACTTTGGTGT

Eif3s6ipR: GGACCTCACCGAGCAGGAGT

Sfrs3F: ACCAGGAAGGAACCCCTTTG

Sfrs3R: CCTTAGGTCCTGCCCAGTTTT

CK334428F: GGTTCCGCTGTCCTCTTTCTC

CK334428R: TGCCTTCCTTGTCTTGGATCTT

Gnb2rs1F:GGTACACTGGGGTGGCTCTG

Gnb2rs1R: GGCCACACTGGCTACCTGAA

Rpl15F: ATGTCAGCCCACGCATTTCT

Rpl15R: GTGGGGCTTTGAGAGTCCTG

Mylc2bF: TGAGCAAGCAAACGCAAGAG

Mylc2bR: ACCGCTTCACAGACGAGGAA

PpiaF: TTGCTGGTCTTGCCATTCCT

PpiaR: ATGTGCCAGGGTGGTGACTT

Slc25a5F: TGATAAAACAGACAAGCCCAGAGA

Slc25a5R: GCAGTCTGGACGCAAAGGAA

Rps15aF: CTGACGTGAAGGGAGCAGGT

Rps15aR: GAATGAATGTTCTGGCGGATG

Pkm2F:TGTTGGGAAAGCAGGTCAGG

Pkm2R: CAGCATCTCTGGGCTTTGCT

Rpl26f: CGGTTGTGCCATTAGCCTTC

Rpl26R: CGTTCGGTCTATGCCCATTC

BG065399F: TCCCTGTGGGCTGTAGATGA

BG065399R: CCGTGTGATTGAGCTGGAGA

H3030G02F: GAGTGCAAGGAGAGCAGTGG

H3030G02R: TTCACGCTTAGTGGGCAGAG

BG066187F: CCAATCAAGACCTTTCCTGCTC

BG066187R: GCCCAAATCCCTGTATTGCT

BG066357F: TGTGCCTCCTACCTACAGACCTT

BG066357R: GTTGGGGTGCTTCAGCTTTC

BG067125F: TCTGATAAATGCACGCATCC

BG067125R: CGGTACAGTGAAACTGCGAAT

BG067313F: TTTGCAGAGGCTAAGGAGGA

BG067313R: TAACAGACCACGCCCAGAAC

Folr4F: AAATGAGGCACCGTAGACTGCT

Folr4R: CCTGATCCCTGCTTCCCTCT

Anxa6 F: GCCACACAGAGCAAGCAAAG

Anxa6 R: CAAGTCCATGAAGGGTGCTG

BG072179F: GAGACTCTGGTGTGGGTTTTCAC

BG072179R: GCACTGGCATTGTCTCTGCTC
