# Supplementary figures and images for: Gene expression profiles during early differentiation of mouse embryonic stem cells
Source: BMC Dev Biol. 2009 Jan 9;9:5. doi: 10.1186/1471-213X-9-5 (PMC2656490; doi:10.1186/1471-213X-9-5)

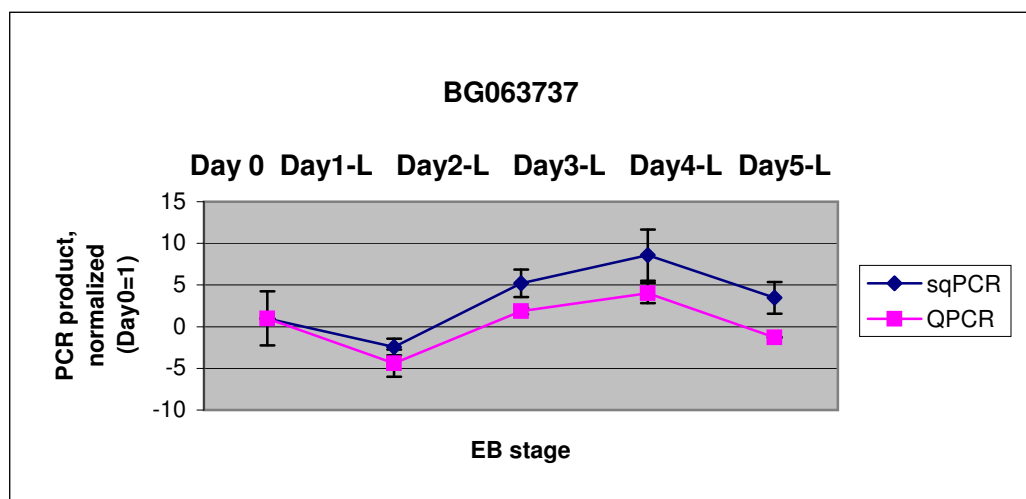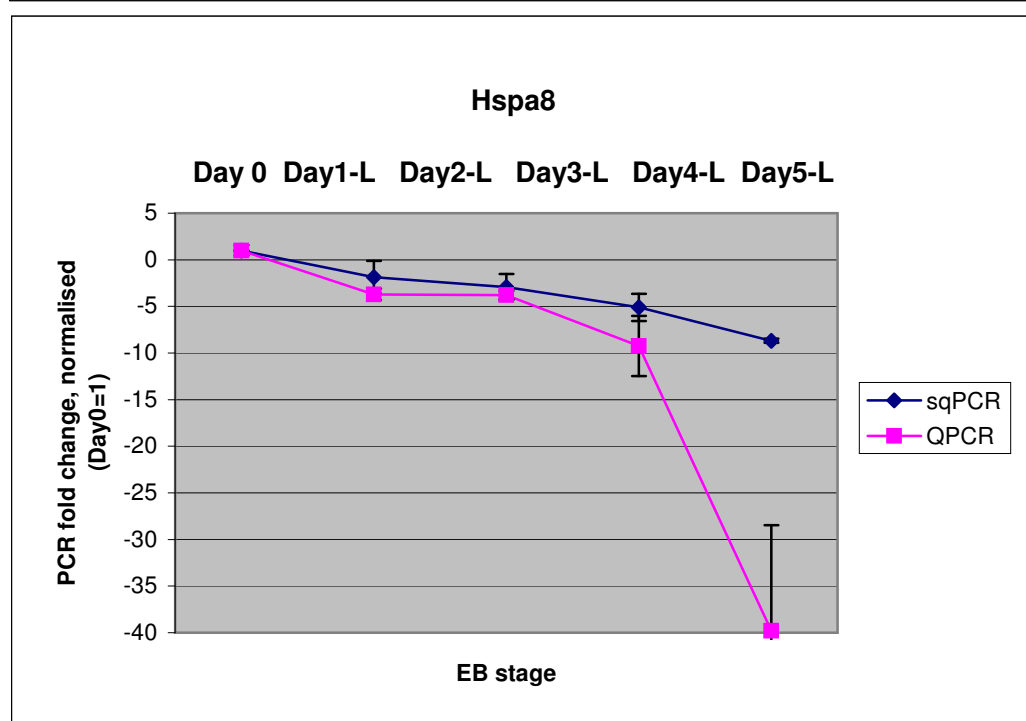

|          |       |          |          |          |          |
|----------|-------|----------|----------|----------|----------|
| BG063737 | Day 0 | Day1-L   | Day2-L   | Day3-L   | Day4-L   |
| sqPCR    | 1     | -2.42564 | 5.200659 | 8.589475 | 3.474219 |
| QPCR     | 1     | -4.37418 | 1.866477 | 4.033209 | -1.28383 |

  

|       |       |          |          |          |          |
|-------|-------|----------|----------|----------|----------|
| Hspa8 | Day 0 | Day1-L   | Day2-L   | Day3-L   | Day4-L   |
| sqPCR | 1     | -1.86613 | -2.91995 | -5.10397 | -8.67887 |
| QPCR  | 1     | -3.7016  | -3.79556 | -9.237   | -39.7826 |

Supplement: Additional file 2 — Q-PCR confirmation of expression patterns of Hspa8 and BG063737 and comparison with semi-quantitative PCR results. Q-PCR confirmation of expression patterns of Hspa8 and BG063737 and comparison with semi-quantitative PCR results. Q-PCR was carried out using an MJ-Research Peltier Thermal Cycler PTC-200 PCR machine and results were analysed using MJ Opticon Monitor 3.1.32 software using previously described methods [39]. Reaction volumes were 25 μl, comprised of 5 μl cDNA, 12.5 μλ PCR mix from the DyNAmo HS SYBR Green qPCR kit (Finnzymes) and 7.5 μl primer mix (25 picomolar). Cycles were as follows: 95°C for 15 mins followed by 34 cycles of 95°C for 30 s, 62°C for 30 s and 72°C for 30 s. Primer sequences are given in Table 1. In order to compare results with those obtained from semi-quantitative PCR, we used Scion Image (Scion Corporation) to measure relative band intensities for 3 repetitions, and processed the figures with reference to the Day 0 sample and the 18S housekeeping gene, such that they were in a similar format to the figures obtained by Q-PCR. While the figures obtained are not identical, the expression patterns seen in terms of trends of differential regulation are very similar; Hspa8 declines in expression from Day 0 while BG063737 dips at day 1-L and peaks at Day 3-L. [file 1471-213X-9-5-S2.pdf]
